# Accuracy of delivered airway pressure and work of breathing estimation during proportional assist ventilation: a bench study

**DOI:** 10.1186/s13613-016-0131-y

**Published:** 2016-04-14

**Authors:** Francois Beloncle, Evangelia Akoumianaki, Nuttapol Rittayamai, Aissam Lyazidi, Laurent Brochard

**Affiliations:** Keenan Research Centre and Li Ka Shing Knowledge Institute, St. Michael’s Hospital, 30 Bond St, Toronto, ON M5B 1W8 Canada; Medical Intensive Care Unit, Hospital of Angers, University of Angers, Angers, France; Department of Intensive Care Medicine, University Hospital of Heraklion, Crete, Greece; Division of Respiratory Diseases and Tuberculosis, Department of Medicine, Faculty of Medicine Siriraj Hospital, Bangkok, Thailand; Institut Supérieur des Sciences de la Santé, Université Hassan 1er, Settat, Morocco; Interdepartmental Division of Critical Care Medicine, University of Toronto, Toronto, ON Canada

## Abstract

**Background:**

Proportional assist ventilation+ (PAV+) delivers airway pressure (*P*_aw_) in proportion to patient effort (*P*_mus_) by using the equation of motion of the respiratory system. PAV+ calculates automatically respiratory mechanics (elastance and resistance); the work of breathing (WOB) is estimated by the ventilator. The accuracy of *P*_mus_ estimation and hence accuracy of the delivered *P*_aw_ and WOB calculation have not been assessed. This study aimed at assessing the accuracy of delivered *P*_aw_ and calculated WOB by PAV+ and examining the factors influencing this accuracy.

**Methods:**

Using an active lung model with different respiratory mechanics, we compared (1) the actual delivered *P*_aw_ by the ventilator to the theoretical *P*_aw_ as defined by the equation of motion and (2) the WOB value displayed by the ventilator to the WOB measured from a Campbell diagram.

**Results:**

Irrespective of respiratory mechanics and gain, the ventilator provided a *P*_aw_ approximately 25 % lower than expected. This underassistance was greatest at the beginning of the inspiration. Intrinsic PEEP (PEEPi), associated with an increase in trigger delay, was a major factor affecting PAV+ accuracy. The absolute value of total WOB displayed by the ventilator was underestimated, but the changes in WOB were accurately detected by the ventilator.

**Conclusion:**

The assistance provided by PAV+ well follows *P*_mus_ but with a constant underassistance. This is associated with an underestimation by the ventilator of the WOB. PEEPi can be a major factor contributing to PAV+ inaccuracy. Clinical recommendations should include using a high trigger sensitivity and a careful PEEP titration.

**Electronic supplementary material:**

The online version of this article (doi:10.1186/s13613-016-0131-y) contains supplementary material, which is available to authorized users.

## Background

Proportional assist ventilation (PAV), described by Younes [1], was the first ventilator mode that introduced the concept of ‘patient-driven’ ventilation: a patient’s effort could influence not only the timing but also the magnitude of the ventilator assistance. This two-way interaction aimed to bypass numerous disadvantages linked to conventional assisted ventilation: patient–ventilator asynchrony, lack of adaptability to changing ventilator demands and loss of normal breathing variability. In critically ill mechanically ventilated patients, PAV has been shown to preserve breathing variability, improve comfort, decrease work of breathing (WOB) and patient–ventilator interaction with the potential to reduce the duration of controlled mechanical ventilation [2–6].

Through automatic calculation of the respiratory system elastance and resistance and using the respiratory system equation of motion, PAV with load adjustable gain factors (PAV+) is able to partition the ventilator (*P*_aw_) and the patient (*P*_mus_) contribution to total pressure of the respiratory system (*P*_tot_) [7–9]. PAV gain, selected by the physician, determines the partition between *P*_mus_ and *P*_tot_. Thus, PAV+ is the only ventilator mode that calculates noninvasively, respiratory mechanics and WOB. This information would be extremely useful to evaluate the efficacy of assistance, to adjust ventilator settings and to assess patient’s respiratory status and take therapeutic decisions [10]. Carteaux et al. recently published a study where they used their own calculations of *P*_mus_ [10]. It was based on the assumption that *P*_aw_ and *P*_mus_ were complementary according to the equation of motion and therefore that the ventilator accurately delivered PAV. Second, whether the values of WOB displayed by the ventilator could be used, instead of calculating *P*_mus_, was not known. Third, we wanted to assess the effect of intrinsic PEEP in case of obstructive lung disorders.

PAV+ estimations of respiratory system mechanics are indirect and hence, based on a number of assumptions which, if not entirely fulfilled, could affect the accuracy and the reliability of *P*_mus_ and WOB calculation and, eventually, the delivered *P*_aw_. The aim of this study was to assess the accuracy of delivered *P*_aw_ and calculated WOB by PAV+ under different conditions in terms of respiratory system mechanics and patient breathing pattern and to examine the factors influencing this accuracy. In the first step, we assessed the accuracy of PAV+ by comparing the actual delivered *P*_aw_ by the ventilator ($P_{{{\text{aw}}_{\text{meas}} }}$) to the theoretical *P*_aw_ as defined by the equation of motion ($P_{{{\text{aw}}_{\text{th}} }}$). Since *P*_aw_ provided by the ventilator is always a proportion of *P*_mus_, errors in *P*_aw_ would reflect errors in *P*_mus_ and, hence, errors in WOB calculation. Therefore, in the second step, we assessed the accuracy of PAV+ WOB calculation by comparing the value displayed by the ventilator to the WOB, estimated by the Campbell diagram.

## Methods

A PB840 ventilator (Puritan-Bennett 840; Covidien, Boulder, USA), which is currently the only ventilator able to deliver PAV+, was used. The ventilator was connected to the lung simulator by a conventional circuit with separate inspiratory and expiratory limbs. The circuit was connected to the lung simulator with the use of a heat–moisture exchanger (HME). Prior to each experiment, the ventilator was calibrated and tested for leaks. All experiments took place using room air [fraction of inspired oxygen (FiO_2_) 21 %].

### Lung model

An Active Servo Lung 5000 (ASL 5000; Ingmar Medical, Pittsburg, PA, USA) was used as described in previous studies [11, 12]. The ASL 5000 is a digitally controlled real-time breathing computerized simulator consisting of a piston moving inside a cylinder. To control the piston’s movement, a microprocessor is programmed with a script driver, which uses a mathematical model of the equation of motion. Instantaneous flow (*V′*)   and *P*_aw_ were measured by flow and pressure sensors at the entrance of the piston, and volume (*V*) is obtained by the integration of *V′* over time. The spontaneous breathing pattern of the lung simulator was determined by the lung model parameters and the inspiratory effort (*P*_mus_). The two main lung model parameters were the compliance *C*_RS_ and the resistance *R*_RS_. The inspiratory effort embodied the breathing rate, effort amplitude, effort slope and inhaled percentage. Thus, a range of respiratory mechanics and inspiratory efforts could be simulated.

### Formulas

Equation of motion of the respiratory system

$$P_{\text{tot}} \, = \, P_{\text{aw}} \, + \, P_{\text{mus}} \, = \, V^{{\prime }} \times R_{\text{RS}} + \, V \times E_{\text{RS}} + {\text{ PEEPt}}$$

where *P*_tot_ is the total pressure applied to the respiratory system, *R*_RS_ and *E*_RS_ the respiratory system resistance and elastance, respectively, *V′* and *V* the instantaneous flow and volume and PEEPt the total positive end-expiratory pressure.

PAV gain represents the proportion which balances the ventilator (*P*_aw_) and the patient (*P*_mus_) contribution to total pressure of the respiratory system (*P*_tot_):

$$P_{\text{aw}} /P_{\text{mus}} \, = \, \% {\text{Gain/}}\left( {100 \, - \, \% {\text{Gain}}} \right).$$

### Design of the experiment

#### Accuracy of *P*_aw_ delivered during PAV+

The ASL 5000 was set at single-compartment model to resemble four respiratory mechanics conditions: normal (*R*_RS_ = 10 cmH_2_O/L/s and *C*_RS_ = 60 mL/cmH_2_O), obstructive (*R*_RS_ = 20 cmH_2_O/L/s and *C*_RS_ = 60 mL/cmH_2_O), restrictive (R_RS_ = 10 cmH_2_O/L/s and *C*_RS_ = 30 mL/cmH_2_O) and mixed obstructive and restrictive (*R*_RS_ = 20 cmH_2_O/L/s and C_RS_ = 30 mL/cmH_2_O). A semi-sinusoidal inspiratory waveform was selected. The inspiratory waveform had a rise time of 30 %, inspiratory holding time of 0 % and releasing time of 15 %. PAV+ was tested under different respiratory mechanics (as described above), gains (30 and 60 %), inspiratory trigger (0.8, 5, and 15 L/min), *P*_mus_ (10 and 15 cmH_2_O) and PEEP levels (0 and 5 cmH_2_O). Various respiratory rates (RR) (10, 15, 20, 25 and 30 breath/min) were examined during obstructive respiratory mechanics to assess the impact of intrinsic PEEP (PEEPi) on delivered *P*_aw_. In total, 24 different conditions were tested.

#### Accuracy of WOB calculation

The ASL 5000 was set as a single-compartment model to resemble three respiratory mechanics: normal (*R*_RS_ = 5 cmH_2_O/L/s, C_RS_ = 60 mL/cmH_2_O), restrictive (*R*_RS_ = 5 cmH_2_O/L/s, *C*_RS_ = 20 mL/cmH_2_O) and obstructive (*R*_RS_ = 20 cmH_2_O/L/s, *C*_RS_ = 60 mL/cmH_2_O). A semi-sinusoidal inspiratory waveform was selected with a rise time of 25 %, inspiratory holding time of 0 % and releasing time of 15 %. Each condition was tested under 2 RR (20 and 30 breaths/min) and across 2 levels of *P*_mus_ (5 and 10 cmH_2_O). The flow triggering was 0.8 L/min and PEEP was 5 cmH_2_O.

Moreover, we also used a real esophageal pressure signal, derived from our patients’ database, to drive the simulator and test PAV+. This signal was tested under normal, restrictive and obstructive conditions.

The aforementioned scenarios were examined at two PAV+ gain levels (30 and 60 %). Therefore, 15 scenarios at two levels of Gain were assessed (30 conditions in total).

#### Data collection

Each test was recorded for 5 min, which was the time period for stabilization of the system. Then the last minute of the recording was selected and analyzed offline.

Data acquisition from ASL 5000 was performed at 128 Hz and stored in a laptop computer for subsequent analysis with AcqKnowledge software (Biopac Systems, Goleta, CA, USA). *V′*, *P*_mus_ and $P_{{{\text{aw}}_{\text{meas}} }}$ curves over time were provided by the ASL 5000. *V* was derived from *V′* integration over time.

PEEPi was estimated as the pressure difference between the *P*_mus_ at the onset of inspiration (defined as the first point where *P*_mus_ started to decrease at end-expiration) and *P*_mus_ at the start of inspiratory *V′*.

#### Accuracy of *P*_aw_ delivered

Inspiratory time (Ti) was defined from the beginning of *P*_mus_ (drop in *P*_mus_ curve) to the end of inspiratory *V′* (Additional file 1: Figure S1). Mean *P*_aw_ during inspiration and *P*_aw_ at 25, 50, 75 and 100 % of Ti were measured. For each parameter, we averaged three cycles not including an occlusion breath or a breath immediately after an occlusion.

Compliance (*C*_vent_) and resistance (*R*_vent_) displayed by the ventilator were recorded, and presented values were the values displayed at the ventilator screen after a stabilization period.

$P_{{{\text{aw}}_{\text{th}} }}$ was calculated from the equation of motion as the following equation:

$${\text{P}}_{{{\text{aw}}_{\text{Th}} }} = \, \left[ {\left( {V /C_{\text{RS}} + V^{{\prime }} \times R_{\text{RS}} } \right) \times {\text{Gain}}} \right] \, + {\text{ total PEEP}}.$$

where total PEEP = the sum of PEEPi above external PEEP and measured external PEEP at the end of expiration.

The difference between the instantaneous mean $P_{{{\text{aw}}_{\text{meas}} }}$ (*i*_meas_) and the mean $P_{{{\text{aw}}_{\text{th}} }}$ (*i*_Th_) were calculated over inspiration (Δ*i*) and at 25, 50, 75 and 100 % of Ti ($\Delta P_{{{\text{aw}}_{25} }} ,\Delta P_{{{\text{aw}}_{50} }} ,\Delta P_{{{\text{aw}}_{75} }} \;{\text{and}}\;\Delta P_{{{\text{aw}}_{100} }}$). They were expressed in percentage of differences related to the $P_{{{\text{aw}}_{\text{th}} }}$ (%Δ*P*_aw_ and %Δ*i*)_._

The percentage of error in measurement of compliance or resistance (%error *C* and %error *R*) was calculated as follows:

$$\% {\text{error }}C = \left( {C_{\text{vent}} - C_{\text{RS}} } \right) /C_{\text{RS}} \times 100, \, \% {\text{error }}R = \left( {R_{\text{vent}} - R_{\text{RS}} } \right) /R_{\text{RS}} \times 100.$$

#### Accuracy of WOB calculation

Data from the ventilator were recorded throughout a specific set of tests with the aid of a software provided by Covidien© and were stored. Data included ventilator settings and calculation of mean *P*_aw_, peak *P*_aw_, *V*, *V′*, minute-ventilation (*V*_*E′*_), RR, Ti, *C*_vent_, *R*_vent_, PEEPi, total PEEP and total displayed WOB in J/L (WOB_displ_). These values were recorded every second and breath by breath. Based on the Campbell diagram, the patient and ventilator WOB were estimated on a breath-by-breath basis. Patient WOB and ventilator WOB were derived by integration of the area plotted between the *P*_mus_–*V* and *P*_aw_–*V* curves, respectively. Total WOB (WOB_real_) was the sum of patient and ventilator WOB. Work of breathing was calculated per liter (WOB per minute divided by *V*_*E′*_) and was expressed in joules per liter (J/L).

The values of total and patient WOB displayed on the PB 840 screen (${\text{WOB}}_{{{\text{tot}}_{\text{displ}} }} ,\,{\text{WOB}}_{{{\text{pt}}_{\text{displ}} }}$) were recorded and those resulting from Campbell (${\text{WOB}}_{{{\text{tot}}_{\text{real}} }} ,{\text{WOB}}_{{{\text{pt}}_{\text{real}} }}$) were calculated. A semiautomated, noncommercially available research software, previously used [13], was used for WOB measurement (SR program, Barcelona).

### Statistical analysis

Variables were expressed as medians (25–75th interquartile range, IQR) or means (±standard deviation, SD). Data were analyzed using nonparametric tests. The relationships between WOB_displ_ and WOB_real_, between %Δ*i* and PEEPi and between %error *C* and %error *R* and PEEPi were evaluated using Pearson’s correlation. Bland–Altman analysis was used to compare the absolute values of WOB_displ_ with those of WOB_real_ which was regarded as the gold standard.

Statistical significance was defined at *p* value <0.05. The statistical analysis was performed using Prism (GraphPad Software, La Jolla, CA, USA).

## Results

### Accuracy of *P*_aw_ delivered during PAV+

#### Effect of different respiratory mechanics and gains

**Fig. 1:**
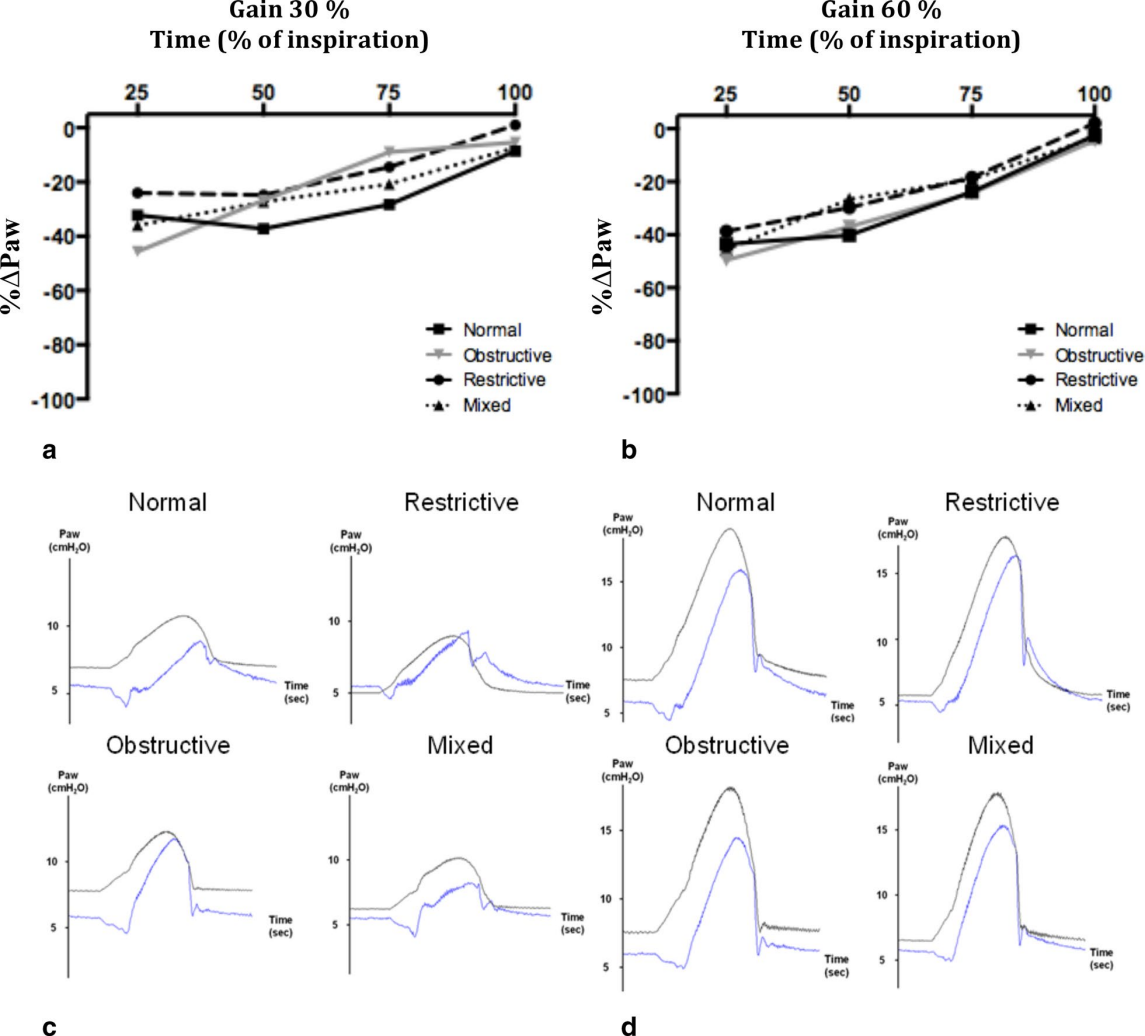
Percentage of difference between measured airway pressure ($P_{{{\text{aw}}_{\text{meas}} }}$) and theoretical airway pressure ($P_{{{\text{aw}}_{\text{th}} }}$) (%Δ*P*_aw_) at 25, 50, 75 and 100 % of inspiration with different lung mechanics with gain 30 % (**a**) and 60 % (**b**). %Δ*P*_aw_ is expressed in percentage of $P_{{{\text{aw}}_{\text{th}} }}$ (%Δ*P*_aw_ = ($P_{{{\text{aw}}_{\text{meas}} }}$ − $P_{{{\text{aw}}_{\text{th}} }}$)/$P_{{{\text{aw}}_{\text{th}} }}$ × 100). Representative tracing of $P_{{{\text{aw}}_{\text{meas}} }}$ and $P_{{{\text{aw}}_{\text{th}} }}$ in 4 respiratory mechanics with gain 30 % (**c**) and 60 % (**d**). *Black lines* $P_{{{\text{aw}}_{\text{th}} }}$ waveforms; *blue lines* $P_{{{\text{aw}}_{\text{meas}} }}$ waveforms. Inspiratory trigger = 5 L/min; muscular pressure = 10 cmH_2_O; PEEP = 5 cmH_2_O; respiratory rate = 20/min. Respiratory mechanics; normal: resistance (*R*) = 10 cmH_2_O/L/s and compliance (*C*) = 60 mL/cmH_2_O; obstructive: *R* = 20 cmH_2_O/L/s and *C* = 60 mL/cmH_2_O; restrictive: *R* = 10 cmH_2_O/L/s and *C* = 30 mL/cmH_2_O; and mixed: *R* = 20 cmH_2_O/L/s and *C* = 30 mL/cmH_2_O

 Irrespective of respiratory mechanics and gain, *i*_meas_ was always lower than *i*_Th_ (Δ*i* and %Δ*i* were −2.9 ± 0.9 cmH_2_O and −25.4 ± 4.6 %, respectively), indicating a lower assistance provided by the ventilator than expected from the equation of motion (Table 1). The magnitude of underassistance was greater at the beginning of the inspiratory cycle and decreased toward the end of inspiration (Fig. 1).

**Table 1:** Measured and theoretical mean airway pressure during inspiration (*i*_meas_ and *i*_Th_) in different respiratory mechanics

Gain (%)	Mechanics	*i*_meas_ (cmH_2_O)	*i*_Th_ (cmH_2_O)	Δ*i* (cmH_2_O)	%Δ*i* (%)
30	Normal	6.6	9.4	−2.8	−29.8
	Obstructive	8.4	10.8	−2.4	−22.2
	Restrictive	7.0	8.5	−1.5	−17.6
	Mixed	6.7	8.8	−2.1	−23.9
60	Normal	9.6	13.7	−4.2	−29.9
	Obstructive	9.5	13.6	−4.1	−30.1
	Restrictive	10.2	13.0	−2.8	−21.5
	Mixed	10.3	13.5	−3.2	−23.7
All conditions		8.5 ± 1.6	11.4 ± 2.3	−2.9 ± 0.9	−25.4 ± 4.6

Difference and percentage of difference between *i*_meas_ and *i*_Th_ were calculated as follows: Δ*i* = *i*_meas_ − *i*_Th_ and %Δ*i* = (*i*_meas_ − *i*_Th_)/*i*_Th_ × 100. Inspiratory trigger = 5 L/min; muscular pressure = 10 cmH_2_O; PEEP = 5 cmH_2_O; respiratory rate = 20/min. Respiratory system mechanics, normal: resistance (R) = 10 cmH_2_O/L/s and compliance (*C*) = 60 mL/cmH_2_O; obstructive: *R* = 20 cmH_2_O/L/s and *C* = 60 mL/cmH_2_O; restrictive: *R* = 10 cmH_2_O/L/s and *C* = 30 mL/cmH_2_O; and mixed: *R* = 20 cmH_2_O/L/s and *C* = 30 mL/cmH_2_O

#### Effect of different triggers, *P*_mus_ and PEEP

**Fig. 2:**
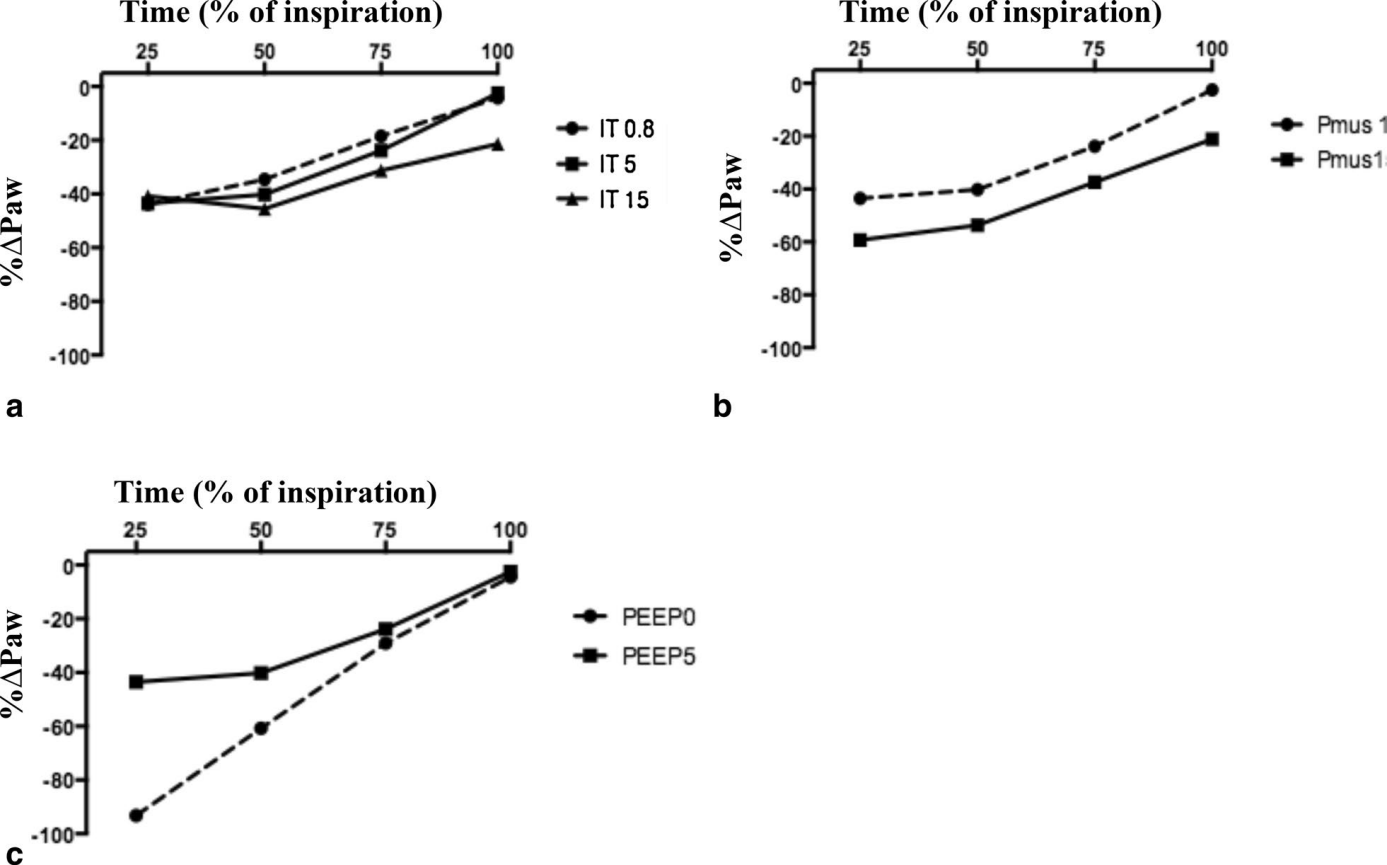
Percentage of difference between measured airway pressure and theoretical airway pressure (%Δ*P*_aw_) at 25, 50, 75 and 100 % of inspiration with different inspiratory trigger (IT) (**a**), muscular pressure (*P*_mus_) (**b**) and positive end-expiratory pressure (PEEP) (**c**) under normal respiratory mechanics. Difference between $P_{{{\text{aw}}_{\text{meas}} }}$ and $P_{{{\text{aw}}_{\text{th}} }}$ is expressed in percentage of $P_{{{\text{aw}}_{\text{th}} }}$ (%Δ*P*_aw_ = ($P_{{{\text{aw}}_{\text{meas}} }}$ − $P_{{{\text{aw}}_{\text{th}} }}$)/$P_{{{\text{aw}}_{\text{th}} }}$ × 100). Resistance = 10 cmH_2_O/L/s; compliance = 60 mL/cmH_2_O; gain = 60 %; and respiratory rate = 20/min; **a** different IT at 0.8, 5 and 15 L/min; *P*_mus_ = 10 cmH_2_O; PEEP = 5 cmH_2_O. **b** Different *P*_mus_ at 10 and 15 cmH_2_O; IT 5 L/min; PEEP = 5 cmH_2_O; **c** different PEEP at 0 and 5 cmH_2_O; IT 5 L/min; *P*_mus_ 10 cmH_2_O

The underassistance of PAV+ was also highlighted under different trigger, *P*_mus_ or PEEP settings in normal respiratory mechanics (Fig. 2; Additional file 2: Tables S1, Additional file 3: Table S2, Additional file 4: Table S3). A higher trigger value (lower sensitivity) led to greater underassistance at the end of inspiration versus a lower trigger (Fig. 2a). A high *P*_mus_ was associated with a greater underassistance during the entire inspiration versus a low *P*_mus_ (Fig. 2b). A decrease in PEEP was associated with a major underassistance at the start of the inspiration (Fig. 2c). These findings were replicated under different trigger, *P*_mus_ or PEEP settings in obstructive and restrictive respiratory mechanics (Additional file 5: Figure S2). Of note, with obstructive respiratory mechanics and trigger = 15 L/min, the ventilator was unable to estimate initial values for *R*_vent_ and *R*_vent_ and the PAV+ mode did not operate.

#### Effect of PEEPi

**Fig. 3:**
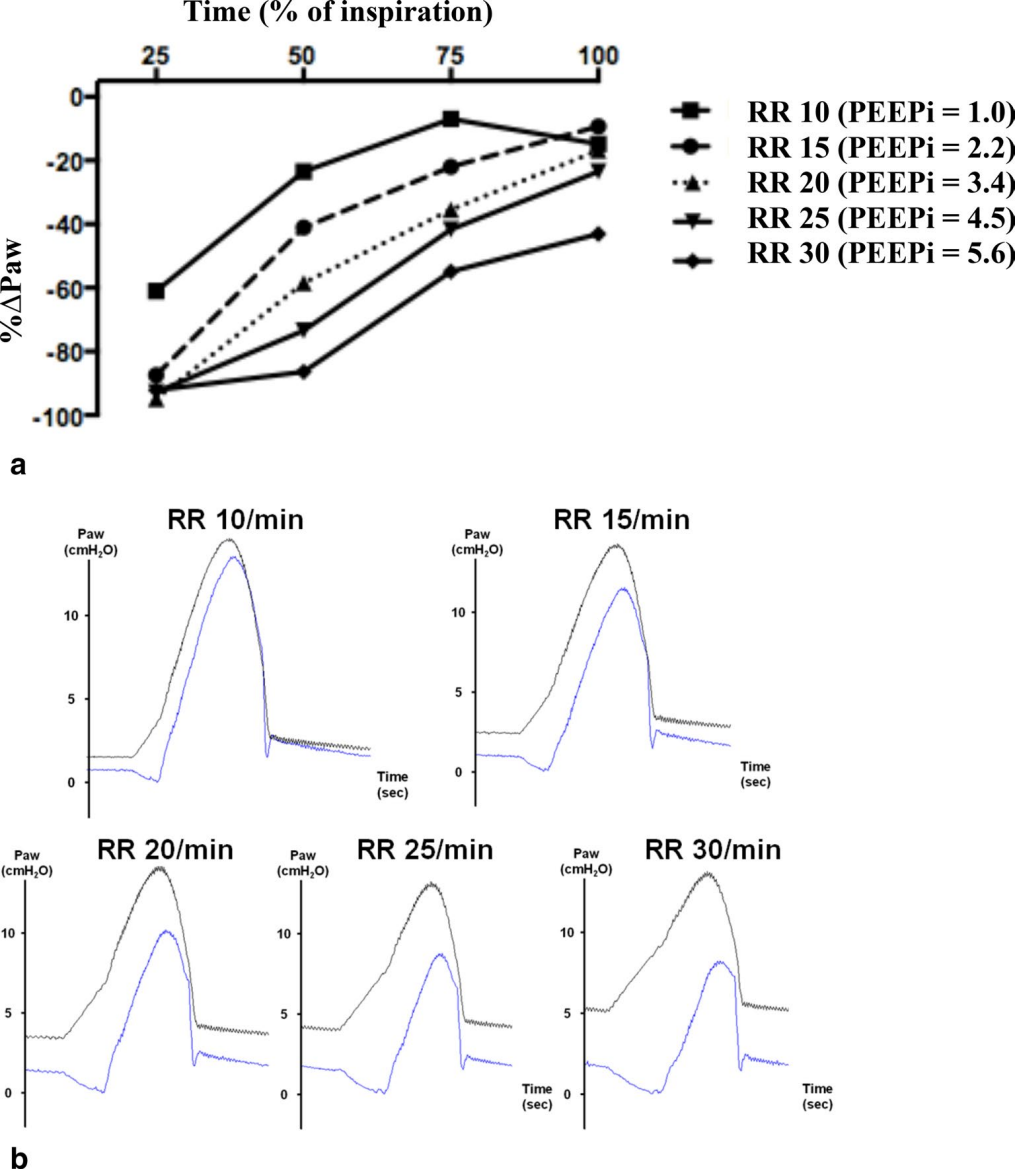
Percentage of difference between measured airway pressure ($P_{{{\text{aw}}_{\text{meas}} }}$) and theoretical airway pressure ($P_{{{\text{aw}}_{\text{th}} }}$) (%Δ*P*_aw_) at 25, 50, 75 and 100 % of inspiration with different respiratory rates in obstructive respiratory mechanics (**a**). Difference between $P_{{{\text{aw}}_{\text{meas}} }}$ and $P_{{{\text{aw}}_{\text{th}} }}$ is expressed in percentage of $P_{{{\text{aw}}_{\text{th}} }}$(% Δ*P*_aw_ = ($P_{{{\text{aw}}_{\text{meas}} }}$ − $P_{{{\text{aw}}_{\text{th}} }}$)/$P_{{{\text{aw}}_{\text{th}} }}$ × 100). Representative tracing of $P_{{{\text{aw}}_{\text{th}} }}$ and $P_{{{\text{aw}}_{\text{meas}} }}$ (**b**). *Black lines* $P_{{{\text{aw}}_{\text{th}} }}$ waveforms. *Blue lines* $P_{{{\text{aw}}_{\text{meas}} }}$ waveforms. Resistance = 20 cmH_2_O/L/s; compliance = 60 mL/cmH_2_O; gain = 60 %; inspiratory trigger = 5 L/min; PEEP = 0 cmH_2_O; and *P*_mus_ = 10 cmH_2_O

To assess the effect of PEEPi on the accuracy of *P*_aw_ delivered by PAV+, the same experiments were replicated under obstructive respiratory mechanics with increasing RR. An increase in RR leading to an increase in PEEPi resulted in a higher %Δ*P*_aw_ during the entire cycle, showing that PEEPi which is associated with an increase in trigger delay affected PAV+ accuracy (Fig. 3; Additional file 6: Table S4). Combining the data from all conditions, PEEPi was correlated with %Δ*i* (Additional file 7: Figure S3). The higher the PEEPi, the lower the pressure assistance the ventilator provided.

#### Measurements of *C*_RS_ and *R*_RS_

In comparison with *C*_RS_, *C*_vent_ was globally slightly overestimated for the low value of compliance (30 mL/cmH_2_O) [34 (IQR 34–36) mL/cmH_2_O] and slightly underestimated for the high value of compliance (60 mL/cmH_2_O) [59 (IQR 63–65) mL/cmH_2_O] (Additional file 8: Figure S4). In comparison with *R*_RS_, *R*_vent_ was always underestimated, irrespective of *R*_RS_ [8.8 (IQR 8.2–8.9) and 15 (IQR 15–16) cmH_2_O/L/s for *R*_RS_ = 10 and 20 cmH_2_O/L/s, respectively] (Additional file 8: Figure S4).

We found a strong correlation between the percentage of error in the measurement of compliance (%error *C*) and PEEPi (*r*^2^ = 0.68, *p* < 0.001, Additional file 9: Figure S5). The association between the percentage of error in the measurement of resistance (%error *R*) and PEEPi was weaker (*r*^2^ = 0.27, *p* = 0.007). This underestimation of compliance in case of high PEEPi should lead to an increase in the assistance delivered by the ventilator in comparison with $P_{{{\text{aw}}_{\text{th}} }}$ (calculated with the actual compliance of the simulator) and thus counteract in part the underassistance observed in PAV+ when PEEPi is high.

### Accuracy of WOB measurements during PAV+

**Fig. 4:**
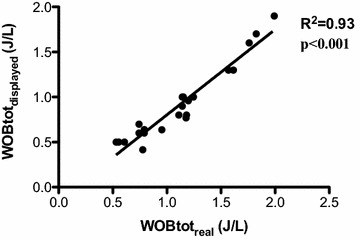
Correlation between the total work of breathing calculated by the ventilator (${\text{WOB}}_{{{\text{tot}}_{\text{displayed}} }}$) and the corresponding calculated by Campbell (${\text{WOB}}_{{{\text{tot}}_{\text{real}} }}$)

**Fig. 5:**
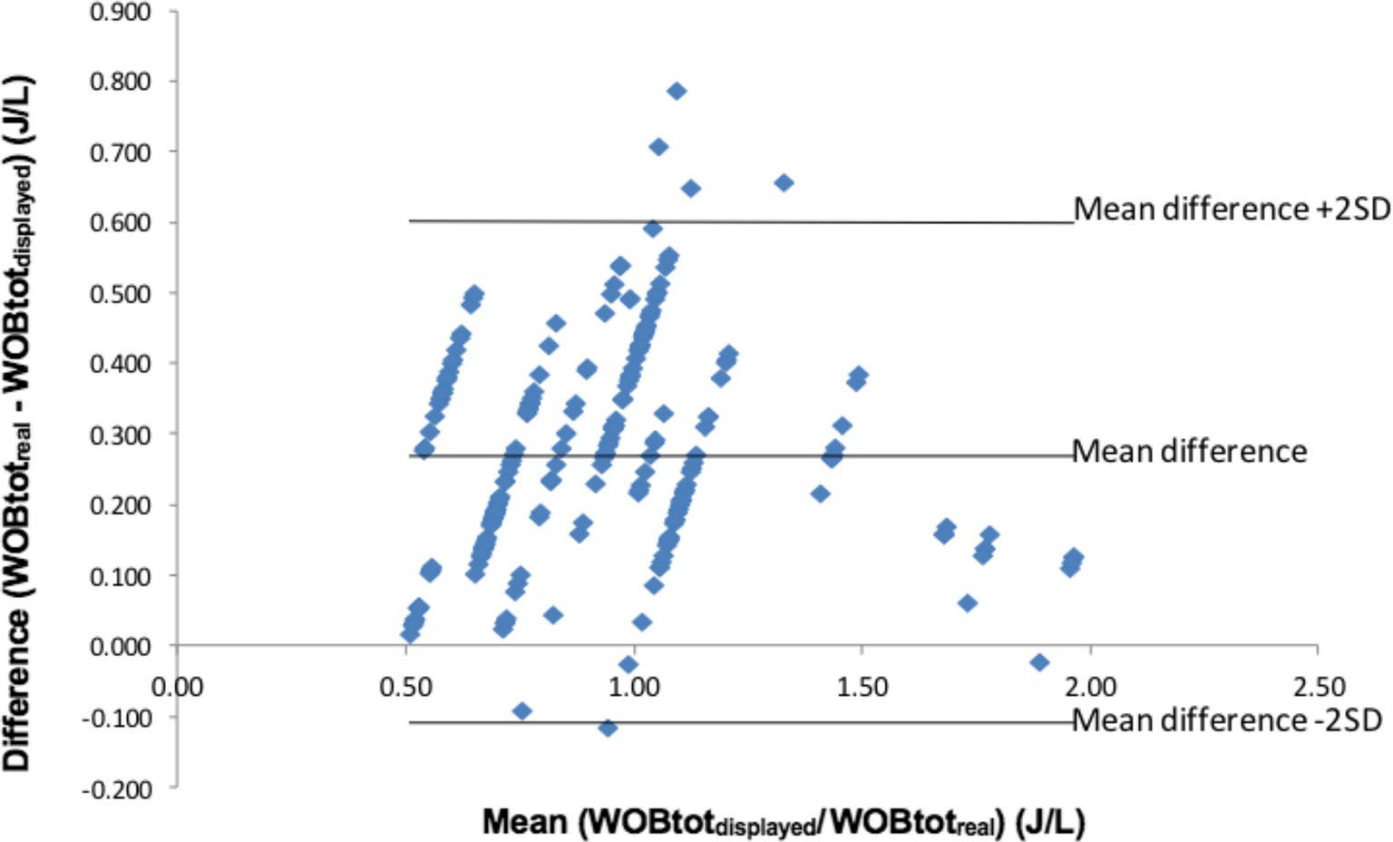
Bland–Altman plot of total inspiratory work of breathing measurements, expressed in J/L, between the two methods compared (Campbell and Ventilator). ${\text{WOB}}_{{{\text{tot}}_{\text{displayed}} }}$, inspiratory work of breathing calculated by the ventilator; ${\text{WOB}}_{{{\text{tot}}_{\text{real}} }}$, inspiratory work of breathing calculated by the Campbell diagram

There was a strong linear correlation between total WOB calculated by the ventilator and total WOB based on the Campbell diagram (*r*^2^ = 0.93, *p* < 0.001, Fig. 4). The Bland–Altman plot, performed to evaluate the accuracy of the absolute values of total WOB calculation, revealed a mean bias of 0.27 J/L, indicating an underestimation of the ${\text{WOB}}_{{{\text{tot}}_{\text{real}} }}$, with a limit of agreement of 0.6 to −0.11 J/L (Fig. 5). The changes in total WOB were accurately detected by the ventilator, but the absolute values of total WOB displayed by the ventilator were underestimated. The linear correlation between the patient’s WOB as calculated by the ventilator and as computed by the Campbell diagram was significant but much weaker than for WOB_tot_ (*r*^2^ = 0.63, *p* < 0.001).

## Discussion

This bench study showed that although the *P*_aw_ delivered by PAV+ reasonably follows the muscular pressure when compared to the theoretical *P*_aw_ (i.e., the pressure that the ventilator should ideally deliver according to the equation of motion of the respiratory system), the ventilator provides a 25 % underassistance irrespective of the respiratory mechanics or ventilator settings. This underassistance is particularly marked when PEEPi is high. Of note even slight PEEPi values lead to dramatic increases in this underassistance (around 40 % for PEEPi values around 4 cmH_2_O).

This inaccuracy in $P_{{{\text{aw}}_{\text{meas}} }}$ compared with $P_{{{\text{aw}}_{\text{th}} }}$ reflects the inaccurate estimation of *P*_mus_ and is thus associated with an underestimation of WOB. Despite the fact that the absolute values of WOB displayed on the ventilator bar graph underestimated the actual WOB, changes in its values accurately reflect measured changes in WOB.

Trigger delay and PEEPi play a pivotal role in this underassistance and in this relative inaccuracy of WOB measurements in PAV+ mode. PEEPi is associated with an increase in trigger delay. On PAV+ mode, once the ventilator is triggered delivered *P*_aw_ is continuously proportional to *P*_mus_, but during the triggering phase no assistance is provided by the ventilator. A delay in the onset of pressurization by the ventilator reduces the correctly assisted fraction of neural inspiratory time and thus the global assistance by the ventilator. Thus, the further increase in trigger delay due to PEEPi leads to a global underassistance. In critically ill patients ventilated in PAV+ mode, Kondili et al. showed that an increase in PEEPi from 0.8 to 3.2 cmH_2_O due to an increase in respiratory workload by chest and abdominal wall compression led to a decrease in the portion of supported inspiratory effort from 86 to 66 % [5]. The conventional pneumatic triggering used in PAV+ ventilation appears as an important limitation especially when compared to the other proportional mode of ventilation (neurally adjusted ventilatory assist), in which the triggering by the electrical activity of the diaphragm (Eadi) is not affected by PEEPi [14, 15]. However, a high PEEPi may also lead to a greater underestimation of *C*_RS_. To estimate the compliance, the ventilator applies a 300-ms pause maneuver at the end of inspiration at random intervals of four to 10 breaths [7]. *P*_aw_ at the end of the occlusion (*P*_aw_, occl) is measured and *C*_RS_ is calculated by the equation of motion (*C* = *V*/(*P*_aw_, occl-totalPEEP)). However, as PB840 cannot detect the actual PEEPi value, this calculated value of *C*_RS_ may be underestimated in case of dynamic hyperinflation [9]. Thus, the assistance provided by the ventilator calculated by using the equation of motion of the respiratory system is increased as a result of this underestimation of *C*_RS_. Overall, PEEPi has two effects on PAV+ accuracy: It is associated with an increase in trigger delay leading to an underassistance, but this effect is in part counterbalanced by the effect on compliance estimation. Of note, this underassistance delivered by PAV+ may prevent the occurrence of runaway phenomena [16].

One of the major advantages of PAV+ is to allow the clinician to assess noninvasively the WOB. We show in this study that the absolute value of total WOB is underestimated by the ventilator. This finding is of particular importance when we consider the way to adjust Gain with a WOB range target [10]. Importantly for clinical practice, the changes in total WOB in a patient are accurately detected by the ventilator.

The main limitation of our study is that it is a bench study. Even if the lung model that we used was set to imitate human spontaneous breathing in different normal and pathological conditions, this does not reproduce the complexity of the control of breathing. Regarding the specific question addressed, however, this does not invalidate our findings, and we simply cannot use these data to comment on the clinical consequences of this.

This study suggests that, in clinical practice, because of the major role of PEEPi in PAV+ inaccuracy, recommendations should include a careful external PEEP titration when PEEPi is suspected. In addition, using a high trigger sensitivity is recommended to reduce the underassistance by PAV+. Following these recommendations, the underassistance has probably a modest clinical impact, whereas WOB values displayed by the ventilator may not be accurate enough to be used to monitor effect of PAV+. However, a clinical study is needed to support these recommendations.

## Conclusion

The PAV+ assistance reasonably well follows *P*_mus_ but provides a constant underassistance of around 25 % on average, especially at the beginning of inspiration. This underassistance is logically associated with an underestimation by the ventilator of the actual total WOB. PEEPi leading to increased trigger delay is a major factor contributing to PAV+ inaccuracy. Clinical recommendations should include using a high trigger sensitivity and a careful PEEP titration when PEEPi is suspected.

## Additional files

10.1186/s13613-016-0131-y Description of measured parameters. (A) Trigger delay time, intrinsic positive end-expiratory pressure (intrinsic PEEP) and inspiratory cycle time (Ti). (B) Theoretical airway pressure (Paw_Th_) and measured airway pressure (Paw_meas_) at 25, 50, 75 and 100 % of Ti.
10.1186/s13613-016-0131-y Measured and theoretical mean airway pressure during inspiration (i_meas_ and i_Th_) with different triggers in different respiratory mechanics.
10.1186/s13613-016-0131-y Measured and theoretical mean airway pressure during inspiration (i_meas_ and i_Th_) with muscular pressure = 15 cmH_2_O in different respiratory mechanics.
10.1186/s13613-016-0131-y Measured and theoretical mean airway pressure during inspiration (i_meas_ and i_Th_) with PEEP=0 cmH_2_O in different respiratory mechanics.
10.1186/s13613-016-0131-y Percentage of difference between measured airway pressure and theoretical airway pressure (%ΔPaw) at 25, 50, 75 and 100 % of inspiration in obstructive and restrictive respiratory mechanics with different inspiratory triggers (IT) (A, B), muscular pressures (Pmus) (C, D) and positive end-expiratory pressure (PEEP) (E, F). Difference between Paw_meas_ and Paw_Th_ is expressed in percentage of Paw_Th_ (%ΔPaw = (Paw_meas_ − Paw_Th_)/Paw_Th_ × 100). Gain = 60 % and respiratory rate = 20/min; respiratory system mechanics, obstructive: resistance (R) = 20 cmH_2_O/L/s and compliance (C) = 60 mL/cmH_2_O and restrictive: R = 10 cmH_2_O/L/s and C = 30 mL/cmH_2_O. (A, B) Different IT at 0.8, 5, and 15 L/min; Pmus = 10 cmH_2_O; PEEP = 5 cmH_2_O. (C, D) Different Pmus at 10 and 15 cmH_2_O. IT 5 L/min; PEEP = 5 cmH_2_O. (E, F) Different PEEP at 0 and 5 cmH_2_O; IT 5 L/min; Pmus 10 cmH_2_O. In obstructive mechanics with IT = 15 L/min, PAV + mode was unable to calculate compliance and resistance and did not operate.
10.1186/s13613-016-0131-y Measured and theoretical mean airway pressure during inspiration (i_meas_ and i_Th_) with different respiratory rates in obstructive mechanics.
10.1186/s13613-016-0131-y Correlation between the percentage of difference between measured and theoretical mean airway pressure during inspiration (%Δi) and intrinsic positive end-expiratory pressure (PEEPi). Each point represents each experimental condition.
10.1186/s13613-016-0131-y Distribution of the values of compliance (A) and resistance (B) measured by the ventilator (C_vent_ and R_vent_) according to the real values of compliance and resistance (C_RS_ and R_RS_). Each point represents each experimental condition.
10.1186/s13613-016-0131-y Correlation between the percentage of error in measurement of compliance (A) or resistance (B) (%error C and %error R) and intrinsic positive end-expiratory pressure (PEEPi).  %error C and %error R were calculated as follows:  %error C = (C_vent_ - C_RS_)/C_RS_ × 100,  %error R = (R_vent_ - R_RS_)/R_RS_ × 100. Each point represents each experimental condition.
