# Supplementary figures and images for: Accuracy of delivered airway pressure and work of breathing estimation during proportional assist ventilation: a bench study
Source: Ann Intensive Care. 2016 Apr 14;6:30. doi: 10.1186/s13613-016-0131-y (PMC4830790; doi:10.1186/s13613-016-0131-y)

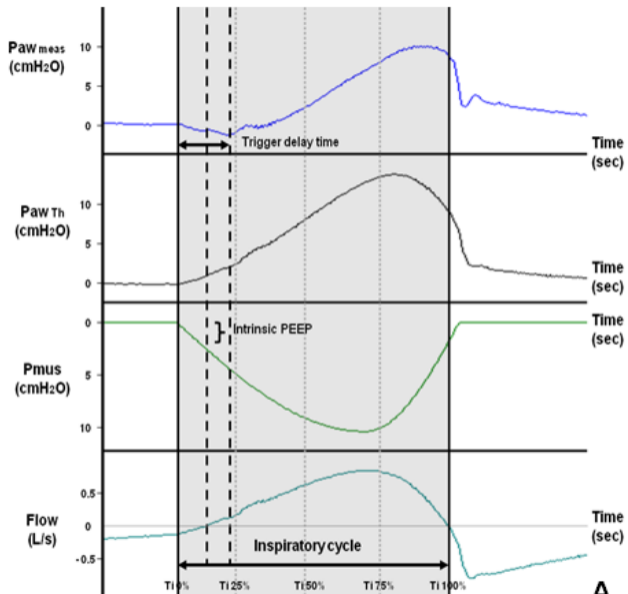

A

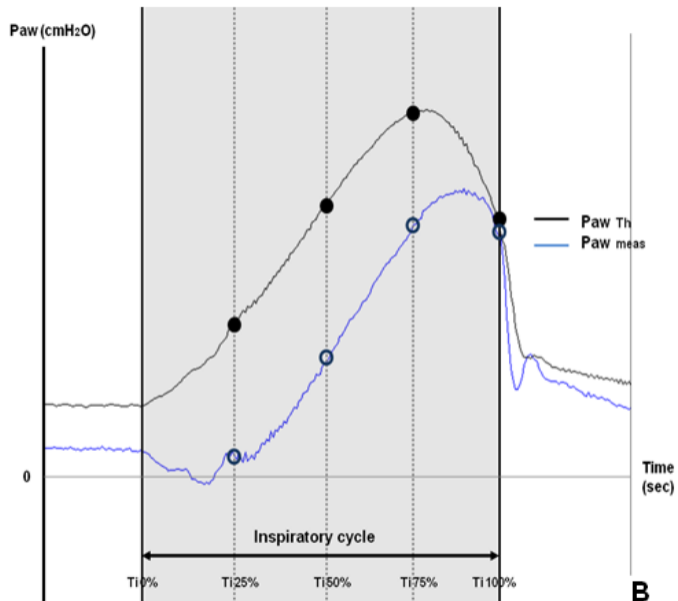

B

Supplement: Supplementary file 1 — 10.1186/s13613-016-0131-y Description of measured parameters. (A) Trigger delay time, intrinsic positive end-expiratory pressure (intrinsic PEEP) and inspiratory cycle time (Ti). (B) Theoretical airway pressure (PawTh) and measured airway pressure (Pawmeas) at 25, 50, 75 and 100 % of Ti. [file 13613_2016_131_MOESM1_ESM.pdf]

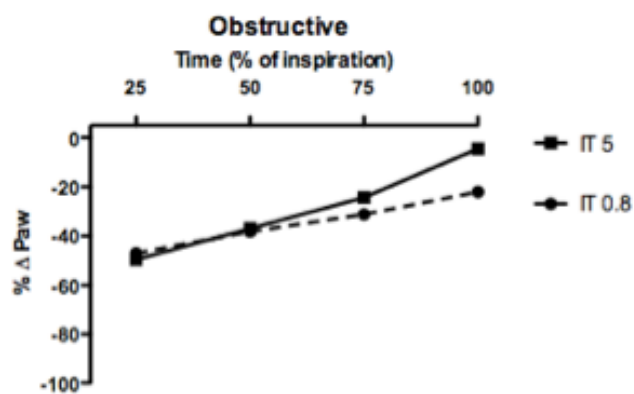

A

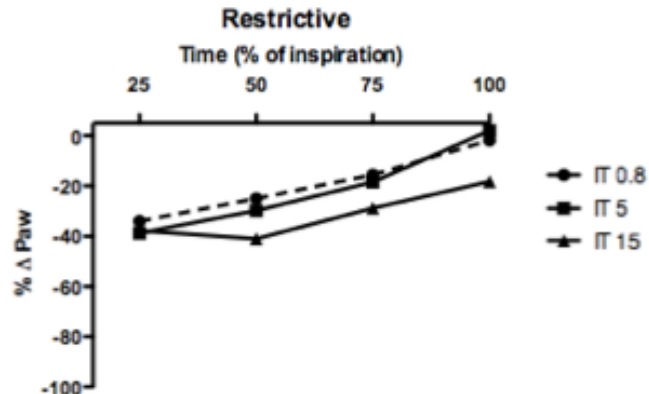

B

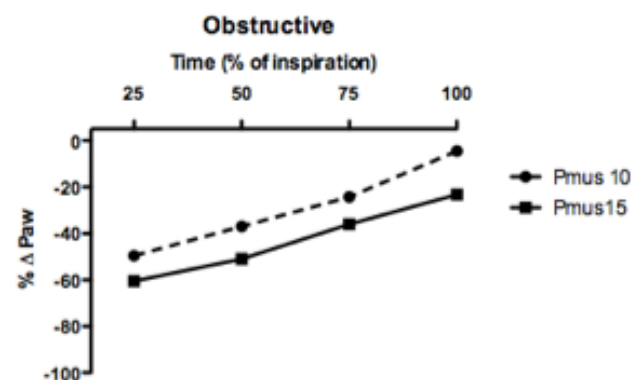

C

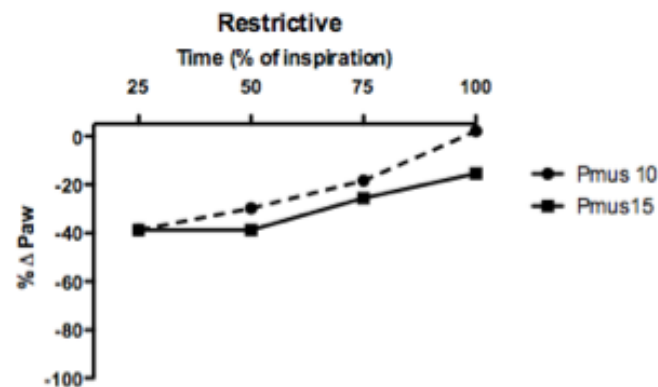

D

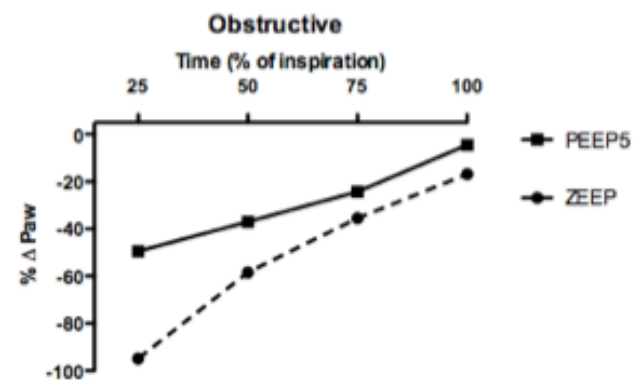

E

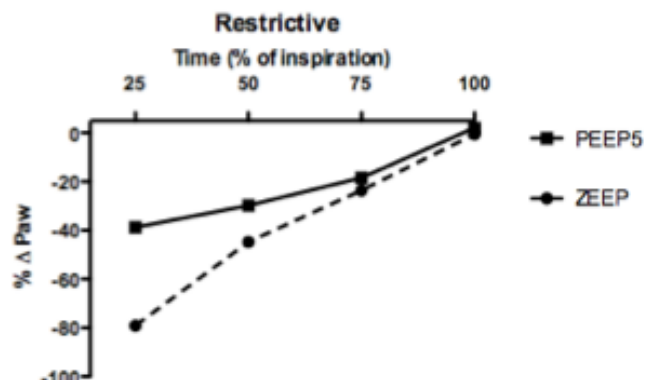

F

Supplement: Supplementary file 5 — 10.1186/s13613-016-0131-y Percentage of difference between measured airway pressure and theoretical airway pressure (%ΔPaw) at 25, 50, 75 and 100 % of inspiration in obstructive and restrictive respiratory mechanics with different inspiratory triggers (IT) (A, B), muscular pressures (Pmus) (C, D) and positive end-expiratory pressure (PEEP) (E, F). Difference between Pawmeas and PawTh is expressed in percentage of PawTh (%ΔPaw = (Pawmeas − PawTh)/PawTh × 100). Gain = 60 % and respiratory rate = 20/min; respiratory system mechanics, obstructive: resistance (R) = 20 cmH2O/L/s and compliance (C) = 60 mL/cmH2O and restrictive: R = 10 cmH2O/L/s and C = 30 mL/cmH2O. (A, B) Different IT at 0.8, 5, and 15 L/min; Pmus = 10 cmH2O; PEEP = 5 cmH2O. (C, D) Different Pmus at 10 and 15 cmH2O. IT 5 L/min; PEEP = 5 cmH2O. (E, F) Different PEEP at 0 and 5 cmH2O; IT 5 L/min; Pmus 10 cmH2O. In obstructive mechanics with IT = 15 L/min, PAV + mode was unable to calculate compliance and resistance and did not operate. [file 13613_2016_131_MOESM5_ESM.pdf]

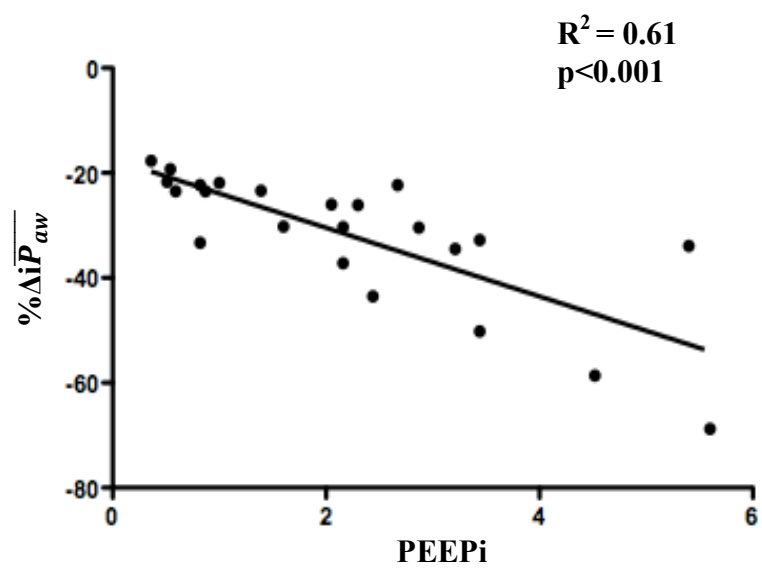

Supplement: Supplementary file 7 — 10.1186/s13613-016-0131-y Correlation between the percentage of difference between measured and theoretical mean airway pressure during inspiration (%Δi) and intrinsic positive end-expiratory pressure (PEEPi). Each point represents each experimental condition. [file 13613_2016_131_MOESM7_ESM.pdf]

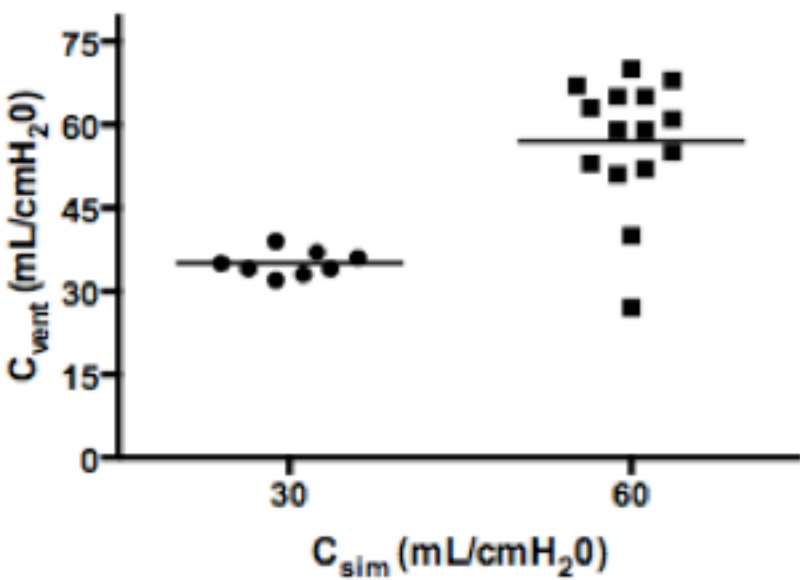

**A**

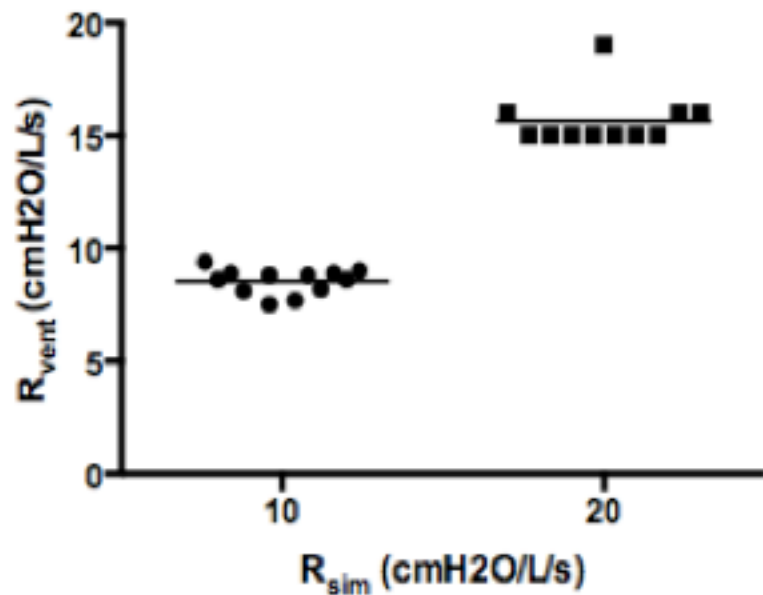

**B**

Supplement: Supplementary file 8 — 10.1186/s13613-016-0131-y Distribution of the values of compliance (A) and resistance (B) measured by the ventilator (Cvent and Rvent) according to the real values of compliance and resistance (CRS and RRS). Each point represents each experimental condition. [file 13613_2016_131_MOESM8_ESM.pdf]

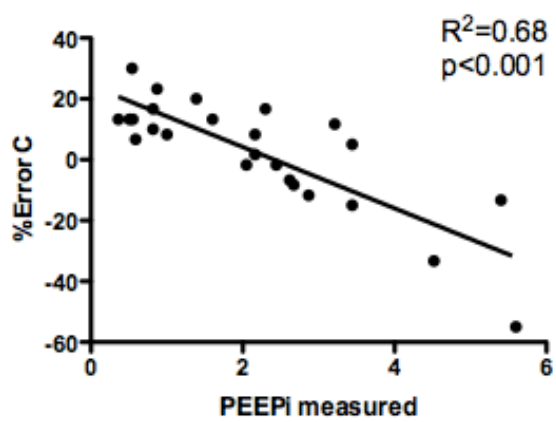

A

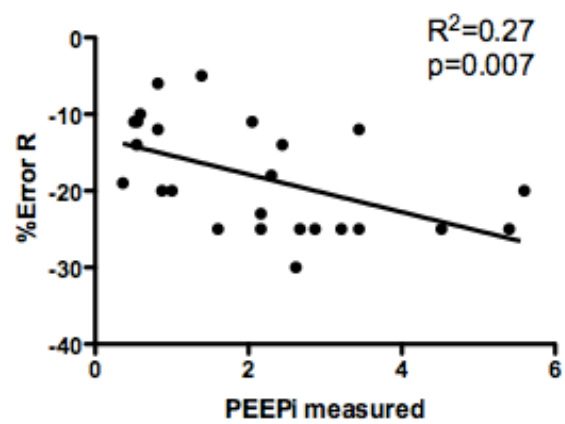

B

Supplement: Supplementary file 9 — 10.1186/s13613-016-0131-y Correlation between the percentage of error in measurement of compliance (A) or resistance (B) (%error C and %error R) and intrinsic positive end-expiratory pressure (PEEPi). %error C and %error R were calculated as follows: %error C = (Cvent - CRS)/CRS × 100, %error R = (Rvent - RRS)/RRS × 100. Each point represents each experimental condition. [file 13613_2016_131_MOESM9_ESM.pdf]
