# Supplementary material for: Accuracy of delivered airway pressure and work of breathing estimation during proportional assist ventilation: a bench study
Source: Ann Intensive Care. 2016 Apr 14;6:30. doi: 10.1186/s13613-016-0131-y (PMC4830790; doi:10.1186/s13613-016-0131-y)
Supplement: Supplementary file 2 — 10.1186/s13613-016-0131-y Measured and theoretical mean airway pressure during inspiration (imeas and iTh) with different triggers in different respiratory mechanics. [file 13613_2016_131_MOESM2_ESM.docx]

**Additional tables**

**Table S1. Measured and theoretical mean airway pressure during inspiration (i_meas_ and i_Th_) with different triggers in different respiratory mechanics.**

| **Inspiratory Trigger (L/min)** | **Mechanics** | **i_meas_ (cm H_2_O)** | **i_Th_**  **(cm H_2_O)** | **Δi (cm H_2_O)** | **%Δi**  **(%)** |
| --- | --- | --- | --- | --- | --- |
| **0.8** | **Normal** | 9.9 | 13.4 | -3.5 | -26.1 |
|  | **Obstructive** | 8.6 | 13.1 | -4.5 | -34.5 |
|  | **Restrictive** | 10.5 | 13.0 | -2.5 | -19.3 |
|  |  |  |  |  |  |
|  | **All mechanics** | 9.6±1.0 | 13.2±0.2 | -3.5±1 | -26.6±.6 |
|  |  |  |  |  |  |
| **10** | **Normal** | 9.5 | 12.8 | -3.3 | -26.0 |
|  | **Obstructive** | - | - | - | - |
|  | **Restrictive** | 9.8 | 12.8 | -3.0 | -23.5 |
|  |  |  |  |  |  |
|  | **All mechanics** | 9.6±0.2 | 12.8±0 | -3.2±0.2 | -24.8±1.8 |

Difference and percentage of difference between i_meas_ andi_Th_ were calculated

as follow _:_ Δi=i_meas_ – i_Th_ and %Δi= (i_meas_ – i_Th_) / i_Th_ × 100). Muscular pressure = 10 cmH_2_O; PEEP = 5 cmH_2_O; respiratory rate = 20/min. Respiratory system mechanics, normal: resistance (R) = 10 cmH_2_0/L/s and compliance (C) = 60 mL/cmH_2_O; obstructive: R= 20 cmH_2_O/L/s and C=60 mL/cmH_2_O and restrictive: R=10 cmH_2_O/L/s and C=30 mL/cmH_2_O. In obstructive mechanics with IT = 15L/min, PAV+ mode was unable to calculate compliance and resistance and did not operate.
