# Supplementary material for: Accuracy of delivered airway pressure and work of breathing estimation during proportional assist ventilation: a bench study
Source: Ann Intensive Care. 2016 Apr 14;6:30. doi: 10.1186/s13613-016-0131-y (PMC4830790; doi:10.1186/s13613-016-0131-y)
Supplement: Supplementary file 3 — 10.1186/s13613-016-0131-y Measured and theoretical mean airway pressure during inspiration (imeas and iTh) with muscular pressure = 15 cmH2O in different respiratory mechanics. [file 13613_2016_131_MOESM3_ESM.docx]

**Table S2. Measured and theoretical mean airway pressure during inspiration (i_meas_ and i_Th_) with muscular pressure = 15 cmH_2_O in different respiratory mechanics.**

| **Pmus**  **(cm H_2_O)** | **Mechanics** | **i_meas_ (cm H_2_O)** | **i_Th_**  **(cm H_2_O)** | **Δi (cm H_2_O)** | **%Δi**  **(%)** |
| --- | --- | --- | --- | --- | --- |
| **15** | **Normal** | 11.8 | 17.6 | -5.8 | -33.0 |
|  | **Obstructive** | 12.1 | 18.3 | -6.2 | -33.9 |
|  | **Restrictive** | 12.7 | 16.4 | -3.6 | -22.0 |
|  |  |  |  |  |  |
|  | **All mechanics** | 12.2±0.5 | 17.4±1.0 | -5.2±1.4 | -27.9±6.6 |

Difference and percentage of difference between i_meas_ and i_Th_ were calculated as follow _:_ Δi = i_meas_ – i_Th_ and %Δi= (i_meas_ – i_Th_) / i_Th_ × 100). Inspiratory trigger = 5 L/min; PEEP = 5 cmH_2_O; respiratory rate = 20/min. Respiratory system mechanics, normal: resistance (R) = 10 cmH_2_O/L/s and compliance (C) = 60 mL/cmH_2_O; obstructive: R= 20 cmH_2_O/L/s and C=60 mL/cmH_2_O and restrictive: R=10 cmH_2_O/L/s and C=30 mL/cmH_2_O.
