# Supplementary material for: Accuracy of delivered airway pressure and work of breathing estimation during proportional assist ventilation: a bench study
Source: Ann Intensive Care. 2016 Apr 14;6:30. doi: 10.1186/s13613-016-0131-y (PMC4830790; doi:10.1186/s13613-016-0131-y)
Supplement: Supplementary file 4 — 10.1186/s13613-016-0131-y Measured and theoretical mean airway pressure during inspiration (imeas and iTh) with PEEP=0 cmH2O in different respiratory mechanics. [file 13613_2016_131_MOESM4_ESM.docx]

**Table S3. Measured and theoretical mean airway pressure during inspiration (i_meas_ and i_Th_) with PEEP=0 cmH_2_O in different respiratory mechanics.**

| **Mechanics** | **i_meas_ (cm H_2_O)** | **i_Th_**  **(cm H_2_O)** | **Δi**  **(cm H_2_O)** | **%Δi**  **(%)** |
| --- | --- | --- | --- | --- |
| **Normal** | 5.2 | 9.2 | -4.0 | -43.5 |
| **Obstructive** | 4.6 | 9.2 | -4.6 | -50.2 |
| **Restrictive** | 5.6 | 8.4 | -2.8 | -33.3 |
|  |  |  |  |  |
| **All mechanics** | 5.1±0.5 | 8.9±0.5 | -3.8±0.9 | -42.3±8.5 |

Difference and percentage of difference between i_meas_ and i_Th_ were calculated as follow _:_ Δi= i_meas_ – i_Th_ and %Δi= (i_meas_ – i_Th_) / i_Th_ × 100). Inspiratory trigger = 5 L/min; muscular pressure = 10 cmH_2_O; respiratory rate = 20/min. Respiratory system mechanics, normal: resistance (R) = 10 cmH_2_O/L/s and compliance (C) = 60 mL/cmH_2_O; obstructive: R= 20 cmH_2_O/L/s and C=60 mL/cmH_2_O and restrictive: R=10 cmH_2_O/L/s and C=30 mL/cmH_2_O.
