# Supplementary material for: Accuracy of delivered airway pressure and work of breathing estimation during proportional assist ventilation: a bench study
Source: Ann Intensive Care. 2016 Apr 14;6:30. doi: 10.1186/s13613-016-0131-y (PMC4830790; doi:10.1186/s13613-016-0131-y)
Supplement: Supplementary file 6 — 10.1186/s13613-016-0131-y Measured and theoretical mean airway pressure during inspiration (imeas and iTh) with different respiratory rates in obstructive mechanics. [file 13613_2016_131_MOESM6_ESM.docx]

| **Respiratory rate** | **i_meas_ (cm H_2_O)** | **i_Th_**  **(cm H_2_O)** | **Δ i (cm H_2_O)** | **%Δ i**  **(%)** |
| --- | --- | --- | --- | --- |
| **10** | 7.1 | 9.1 | -2.0 | -22.0 |
| **15** | 5.6 | 9.0 | -3.3 | -37.8 |
| **20** | 4.6 | 9.2 | -4.6 | -50.0 |
| **25** | 3.9 | 9.4 | -5.5 | -58.5 |
| **30** | 3.1 | 9.8 | -6.7 | -68.4 |

**Table S4. Measured and theoretical mean airway pressure during inspiration (i_meas_ and i_Th_) with different respiratory rate in obstructive mechanics.**

Difference and percentage of difference between i_meas_ and i_Th_ were calculated as follow _:_ Δi = i_meas_ – i_Th_ and %Δi= (i_meas_ – i_Th_)/ i_Th_ × 100). Inspiratory trigger = 5 L/min; muscular pressure = 10 cmH_2_O; PEEP = 0 cmH_2_O; resistance = 20 cmH_2_O/L/s and compliance = 60 mL/cmH_2_O.
